# Clinical Study on the Prevention of Oxaliplatin-Induced Neurotoxicity with Guilongtongluofang: Results of a Randomized, Double-Blind, Placebo-Controlled Trial

**DOI:** 10.1155/2013/541217

**Published:** 2013-11-13

**Authors:** Yunfang Liu, Guangying Zhu, Li Han, Jie Liu, Ting Ma, Huiming Yu

**Affiliations:** ^1^Shandong University Medical School, Jinan, Shandong 250012, China; ^2^Cancer Hospital, Peking University, Beijing 100142, China; ^3^Dalian University, Dalian, Liaoning 116023, China; ^4^Shandong Tumor Hospital & Institute, Jinan, Shandong 250117, China; ^5^Shandong University of Traditional Chinese Medicine, Jinan, Shandong 250014, China; ^6^Department of Radiotherapy, Capital Medical University Affiliated Beijing Chao-yang Hospital, No. 8, Gongti South Road, Beijing 100020, China

## Abstract

*Objective*. Oxaliplatin-induced peripheral neurotoxicity continues to be a kind of frequent dose-limiting toxicity for many cancer patients. This study evaluated the preventive effects of Guilongtongluofang on peripheral neurotoxicity induced by oxaliplatin in patients with colorectal tumor. *Patients and Methods*. From May 2007 to May 2011, we conducted a randomized, double-blind, placebo-controlled trial. 120 patients of colorectal cancer treated with adjuvant oxaliplatin-based chemotherapy were randomly enrolled into the trial group and the control group. The trial group received Guilongtongluofang (at a dose of 200 mL once a day) from 3 days prior to chemotherapy. The control group received a placebo from 3 days prior to chemotherapy. Every 2-week cycle, neurotoxicity was evaluated using numeric rating scale for pain intensity and experienced relief. The primary endpoint was efficacy measurement which included oxaliplatin-induced neurotoxicity and tumor response. The differences of side effects between the two groups were also analyzed. *Results*. The percentage of grades 1-2 neurotoxicity was significantly lower in the trial group than that in the control group (13.3% versus 20.0%; *P* < 0.05) after two cycles of treatment. The difference of the percentage of neurotoxicity between the two groups was significant after six cycles (51.7% versus 70.0%; *P* < 0.05). Significant difference for the mean time to the development of grade 1+ neurotoxicity was found between the two groups (9.4 w in the trial group versus 6.5 w in the control group, *P* < 0.05). The cumulative incidence of grade 1 or more sensory neurotoxicity was significantly lower in the trial group than that in the control group (*P* < 0.05). No significant differences of tumor response rate were found between the two groups the trial group and the control group. No significant difference was found between the trial group and the control group (all *P* > 0.05). *Conclusion*. This study provides evidence that Guilongtongluofang is a promising drug for the prevention of oxaliplatin-induced neurotoxicity in patients with colorectal cancer, and it does not reduce the efficacy of oxaliplatin.

## 1. Introduction

Oxaliplatin is the third-generation platinum-based anticancer drug and is a useful drug in colorectal cancer therapy. Especially, oxaliplatin combined with 5-fluorouracil(5FU)/leucovorin (FOLFOX) has emerged as the standard of care in adjuvant treatment and in first-line and second-line therapy of advanced-stage colorectal cancer [1, 2]. Its overall safety profile is good, but neurotoxicity is the main adverse effect and is a kind of dose-limiting toxicity. It often leads to chemotherapeutic dosage reduction, treatment delay, and treatment discontinuation [2, 3], even when the patient is still responding to the drug.

Oxaliplatin-induced neurotoxicity can be divided into two distinct syndromes. The first one is a unique syndrome of acute, transient peripheral nerve hyperexcitability, which is unique among the platinum complexes studied to date [4]. Patients may experience cold-sensitive paresthesias and dysesthesias of the hands and feet, as well as larynx and jaw. This kind of neurotoxicity usually occurs shortly after the infusion of oxaliplatin and at low total cumulative dose. Acute neurotoxicity occurs frequently, with the incidence varying from 81.5% to 98%, and is often induced by cold exposure [5]. It is always reversible and does not require discontinuation of therapy. The second syndrome is a peripheral sensory neurotoxicity occurring mainly in the distal extremities with symptoms similar to those caused by cisplatin. Development of this syndrome is related to the cumulative dose, generally becoming a clinical problem when the cumulative dose approximates 800 mg/m^2^. It is reversible, but it may last for several months and lead to discontinuation of treatment [6, 7].

Several strategies have been proposed to prevent or treat oxaliplatin-induced neurotoxicity, such as gabapentin, calcium-magnesium infusions, antiepileptic drugs like carbamazepine, amifostine, and glutathione. However, there were no randomized trials which had demonstrated a prophylactic or therapeutic effect of these agents on oxaliplatin cumulative neurotoxicity. Guilongtongluofang, a traditional Chinese medicine, is composed of ramulus cinnamomi, radix astragali, earthworm, safflower, radix angelicae sinensis, ligusticum, spatholobus, radix paeoniae alba, rhizoma curcumae, and licorice. All the herbs added increased the function of warming and activating yang to promote blood circulation to relieve from symptoms such as numbness and cold sensation in patients. 

We hypothesized that Guilongtongluofang might have effect on the prevention of oxaliplatin-induced neurotoxicity. This study was therefore designed to determine the preventive effects of Guilongtongluofang on peripheral neurotoxicity induced by oxaliplatin in patients with colorectal tumor.

## 2. Materials and Methods

### 2.1. Plant Material and Preparation of the Extract

The plant material was purchased from the Beijing Medicinal Material Company (Beijing, China). Guilongtongluofang was composed of ramulus cinnamomi 9 g, earthworm 12 g, radix astragali 30 g, safflower 10 g, radix angelicae sinensis 12 g, ligusticum 12 g, spatholobus 30 g, radix paeoniae alba 30 g, rhizoma curcumae 9 g, and licorice 6 g. The aqueous extract was prepared by the following processes: each dose of the dried herbs was twice decocted in water to 100 mL, and then the 200 mL was mixed and divided into two potions to be taken twice daily.

### 2.2. Patient Population

Consecutive patients with colon or rectum cancer were enrolled in this prospective study from the chemotherapy department of Weifang Hospital of Traditional Chinese Medicine during the period from May 2007 to May 2011. Criteria for enrollment include (1) ≥18 years of age; (2) Eastern Cooperative Oncology Group performance status of 0 to 2; (3) normal bone marrow function (leukocyte count > 4,000/L, platelet count > 100,000/L), liver function (serum bilirubin < 1.5 mg/dL), renal function (creatinine < 1.5 mg/dL), and cardiac function (stable heart rhythm, no active angina, and no clinical evidence of congestive heart failure); (4) life expectancy ≥ 6 months; (5) no preexisting peripheral neurotoxicity from any cause. Pregnant or nursing women were not eligible for this study. Patients with diabetic neuropathy were also excluded. Concomitant use of anticoagulants, platelet aggregation inhibitors, opioids, anticonvulsants, tricyclic antidepressants, and other neuropathic pain medication agents was also prohibited. Informed consent was obtained from all participants, and this study was approved by the local ethics committee.

Using a prospective, randomized, placebo-controlled, double-blind design, 120 patients were randomly assigned into the trial group (60 patients) and the control group (60 patients). There are 83 males and 37 females, with a median age of 52.5 years. Group assignment for all subjects was determined using a random table prior to initiation of the study. The sequence of assignments was unknown to any of the investigators. Each assignment was kept in a sealed envelope, and the order in numeric number was shown on the outside of the envelope. Thus, the orders could not be changed. Envelopes were arranged in order. The principal investigator generated this random selection a few months before recruiting the first subject. No significant differences in gender, age, physical condition, and clinical stage of disease between the two groups were found.

### 2.3. Treatment

All of the patients were given FOLFOX4 chemotherapy for six cycles. The chemotherapeutic regimen consisted of oxaliplatin 85 mg/m^2^ on day 1, given as a 3-hour infusion in 250 mL of dextrose 5%, concurrent with 6-S-stereoisomer of leucovorin 200 mg/m^2^ as a 2-hour infusion followed by fluorouracil 400 mg/m^2^ and 24-hour infusion of fluorouracil 2400 mg/m^2^/d for 2 consecutive days. Then, two weeks later, the patients were given the same chemotherapeutic regimen for 2 consecutive days again. Patients who finished twice chemotherapeutic regimen were defined as one cycle. 

The trial group was given Guilongtongluofang from 3 days prior every chemotherapy and the herb treatment was administered for consecutive 10 days. One dose of Guilongtongluofang was taken every day. The control group was given placebo seen as Guilongtongluofang in the same way. 

### 2.4. Study Endpoints

The primary study endpoint was efficient measurement which included oxaliplatin-induced neurotoxicity and tumor response.

Oxaliplatin-induced neurotoxicity was assessed after every two cycles using the National Cancer Institute's (NCI) common toxicity criteria (CTC) as follows: grade 1: paresthesia and/or dysesthesia (induced by cold) with complete regression within one week; grade 2: paresthesia and/or dysesthesia with complete regression within 14 days; grade 3: paresthesia and/or dysesthesia with incomplete regression at day 14; grade 4: paresthesia and/or dysesthesia with functional consequences [8]. Complete neurological examinations were performed at baseline and after two, four, and six cycles of treatment, respectively. An experienced neurologist evaluated the data to assess possible between-group differences in electrophysiological function. 

Treatment response was assessed according to World Health Organization criteria based on a CT scan 2 months after the completion of treatment. A complete response was defined as the disappearance of all known disease for at least 4 weeks. A partial response required a reduction of at least 50% in the size of the tumor for at least 4 weeks. Progressive disease was defined as an increase of 25% or more in the size of the tumor, and stable disease was defined as no change or less than 50% reduction or more than 25% increase.

The secondary endpoint was safety, and the number of participants discontinued to treatment. All patients underwent followup office visits every two weeks until 2 months after the completion of treatment. At each followup, blood routine, transaminases, blood urea nitrogen (BUN), and creatinine were measured, and the incidence and severity of various side effects (i.e., diarrhea, nausea, vomiting, headache, dizziness and abdominal pain, etc.) which may be associated with treatment were monitored.

### 2.5. Statistical Analysis

With the published event rates for our primary endpoint, we estimated the number of subjects required for the study to have >80% power (*α* = 0.05) to detect an absolute 30% reduction in the incidence of the endpoint. Continuous data were expressed as mean ± SD, and discrete data were given as counts and percentages. Pearson Chi-square test was used to compare categorical variables and the difference of percentage (rate) of neurotoxicity. Proportion of patients with grade 1 to grade 4 neurotoxicity was also compared with a two-sided Fisher's exact test. All data were analyzed using SPSS version 17.0 for windows. Any *P* value given is two sided and subjects to a local significant level of 5%.

## 3. Results

### 3.1. Patient Characteristics

Patient characteristics are shown in Table 1. There were no significant between-group differences in age, gender, performance status, location of primary tumor, histological differentiation, and sites of distant metastasis. All the patients completed at least six treating cycles and were qualified for analysis. There were no drop-out cases.

### 3.2. Oxaliplatin-Induced Neurotoxicity

As shown in Table 2, after two cycles of treatment, 8 patients in the trial group and 11 patients in the control group had grades 1-2 neurotoxicity, the percentage of neurotoxicity was significantly lower in the trial group than that in the control group (13.3% versus 20.0%; *P* < 0.05). After four cycles, 14 (23.3%) patients in the trial group and 18 (30.0%) patients in the control group experienced grades 1-2 neurotoxicity. The difference of the percentage of neurotoxicity between the two groups was nearly significant after four cycles of treatment (28.3% versus 43.3%; *P* = 0.05) and significant after six cycles (51.7% versus 70.0%; *P* < 0.05). 

**Figure 1:**
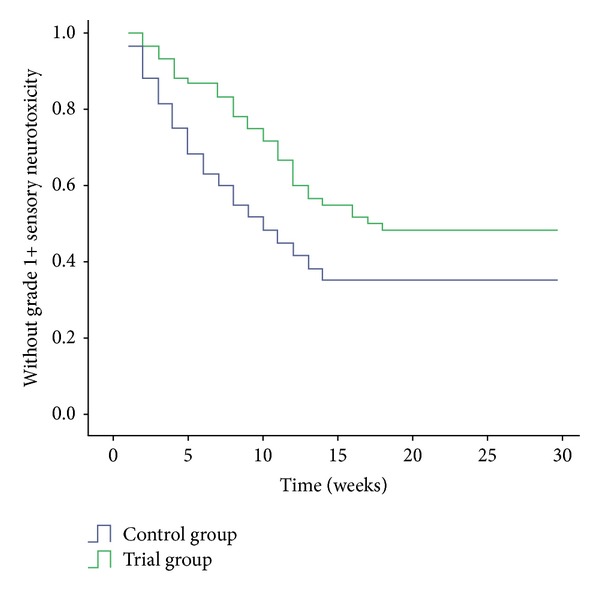
Time to grade 1 or more sensory neurotoxicity in patients treated with or without Guilongtongluofang.

In addition, the onset of grade 1 or more sensory neurotoxicity was much later in patients who received Guilongtongluofang. Data regarding the time to grade 1+ sensory neurotoxicity are illustrated in Figure 1. Significant difference for the mean time to the development of grade 1+ neurotoxicity was found between the two groups (9.4 w in the trial group versus 6.5 w in the control group, *P* < 0.05). 

**Figure 2:**
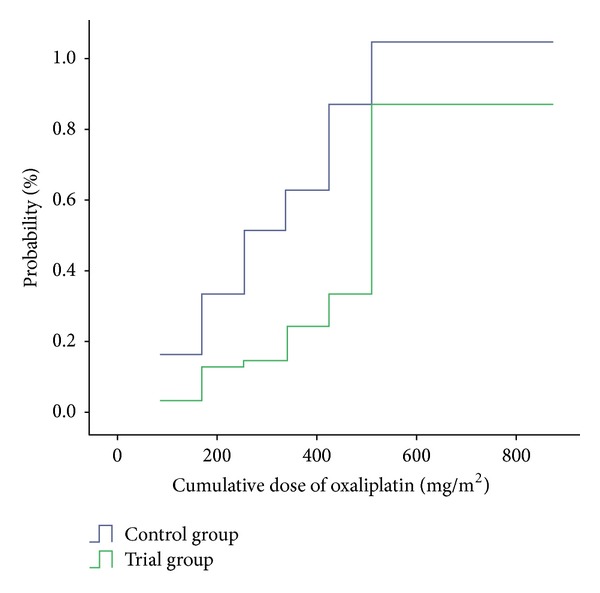
Probability of sensory neurotoxicity by cumulative dose of oxaliplatin in patients treated with or without Guilongtongluofang.

The incidence of grade 1 or more sensory neurotoxicity increased with the increasing cumulative dose of oxaliplatin (Figure 2). The cumulative incidence of grade 1 or more sensory neurotoxicity was significantly lower in the trial group than that in the control group (*P* < 0.05). The median cumulative oxaliplatin doses are 510 mg in the trial group and 255 mg in the control group, respectively.

### 3.3. Tumor Response

All patients completed six cycles of treatment, and the overall response rates (complete response and partial response) were 43.3% in the trial group and 35.0% in the control group, respectively. Two patients in the trial group and two patients in the control group had a complete response, respectively. No significant differences of tumor response rate were found between the two groups (Table 3).

### 3.4. Adverse Events

Nonneurologic adverse events which may be associated with the treatment are listed in Table 4. No significant difference was found between the trial group and the control group (all *P* > 0.05).

## 4. Discussion

Oxaliplatin has become an integral component of chemotherapeutic regimens for the treatment of colon or rectum cancer [9, 10]. Neurotoxicity is the most severe and dose-limiting cumulative toxicity resulting from oxaliplatin therapy [11]. The main neurotoxicity was cold-induced paresthesia after the use of oxaliplatin, which included hyperesthesia, chill, numbness of the hands and feet, electrified sensation, formication, foreign body sensation, and pain that might be exacerbated by exposure to cold.

The main target organ of platinum-based preparations with peripheral neurotoxicity is the dorsal root ganglion, which is consistent with the platinum accumulation studies [12]. The cause of neurotoxicity induced by oxaliplatin is still poorly understood, as demonstrated by the lack of efficacy of the various agents listed previously [13]; therefore, many other theories and drugs are currently being explored. One theory is the connection between free radicals and chemotherapy as a possible cause of neurotoxicity. Free radicals are highly reactive compounds with one or more unpaired electron. Some other neuromodulatory agents have already been tested in patients with oxaliplatin-induced neurosensory toxicity. In a pilot, single-arm study, calcium gluconate, and magnesium sulfate infusions, prior and after oxaliplatin, seemed to be active against acute symptoms [14]. Unfortunately, the prospective evaluation of this treatment was interrupted because of a lower tumor response rate in the Ca/Mg arm [15]. Other studies have proved that Ca/Mg salts could decrease the incidence of oxaliplatin-induced acute and cumulative neurotoxicity and thus enhance patients' tolerance to oxaliplatin, without significantly altering the efficacy of chemotherapy [16, 17]. 

In this study, we also proved the effects of Guilongtongluofang on preventing acute and chronic oxaliplatin-induced neurotoxicity. 8 patients in the trial group and 11 patients in the control group appeared grades 1-2 neurotoxicity after two cycles of chemotherapy. After four cycles, and especially six cycles oxaliplatin-induced neurotoxicity gradually increased with increasing dose. It showed that the neurotoxicity was dose-dependent. After four cycles, 14 patients (23.3%) in the trial group and 18 patients (30.0%) in the control group experienced grades 1-2 sensory neurotoxicity. The difference of the incidence of neurotoxicity between the two groups was significantly different after six cycles of treatment. As for tumor response, the response rate was 43.3% in the trial group and 35.0% in the control group, no significant difference was found between the two groups.

Most scholars considered [18] that the platinum concentrations are greater in the dorsal root ganglion followed by dorsal root and peripheral nerves. The accumulation of oxaliplatin or metabolites oxalate in the dorsal root ganglion is attributed to increasing the outflow and slowing in-flow of Na^+^, so it increases the negative values of the membrane potential and weakens action potential, leading to high sensitivity and excitability of the peripheral nerve. Oxaliplatin can also adjust apoptosis and the balance of cell cycle. It can interact with mitochondrial DNA, lead to oxidative stress, and increase p53 activity and mitochondrial release of cytocrome-c pathway, independent of Fas receptor activation, as well as activation of p38 and ERK1/2 [19]. Scuteri et al. [20] found that the validity of MAPKs is the target of neuroprotective therapies during chemotherapeutic treatment. One method of avoiding oxaliplatin-induced neurotoxicity is kept away from cold drinks and cold objects. Clinicians should also pay close attention to the oxaliplatin cumulative dose and dosing interval. Patients affected are usually those who received doses ≥540 mg/m^2^ or over 4 cycles of therapy [21].

 According to the modern pharmacological research, the main component of *Astragalus* are amino acids, flavonoids, and trace elements, which cannot only improve cell tolerance to hypoxia, antioxidant capacity, and scavenging free radical but also dilate blood vessels, improve the blood supply of cardiac muscle, and reduce the viscosity of blood [22, 23]. It is also useful to resist tumor and improve immunity [24]. Earthworm, safflower, and chuanxiong can dilate the blood vessels, improve microcirculation and blood flow, inhibit platelet aggregation, and prevent thrombosis [25]. Ramulus cinnamomi and radix paeoniae alba are useful in dilating blood vessels and promoting and adjusting blood circulation [26]. Rhizoma curcumae is used for invigorating Qi to dissipate blood stasis and relieve pain [27]. All the herbs together can increase the function of warming Yang, flowing Qi, and activating blood circulation by benefiting vital energy and nourishing blood. All these above bring about dissipating blood stasis to dredge the collateral, detoxicating and resolving stagnation of pathogens and activating blood circulation to dissipate blood stasis. Thus, Guilongtongluofang has the function of activating blood circulation and repairing nerve injury, which leads to the prevention of peripheral neurotoxicity. 

There are some limitations in our study. First, all patients underwent follow-up office visits every two weeks until 2 months after the completion of treatment, and hence a long-term study is obviously needed to further confirm our results. Second, clinical findings were used as the endpoints in our study, whereas a hard clinical endpoint such as mortality should be used in a large sample of patients in a future study.

In summary, the application of FOLFOX4 treatment with Guilongtongluofang in advanced colorectal cancer cannot only reduce the incidence of neurotoxicity but also improve the quality of life in patients. It does not reduce the efficacy of the treatment or increase the toxicity of chemotherapy. 

## 5. Conclusion

Guilongtongluofang can safely decrease the incidence of severe neurotoxicity induced by FOLFOX4 regimen, without reducing the efficacy of the treatment or increasing the side effects. Therefore, Guilongtongluofang is useful in preventing oxaliplatin-induced neurotoxicity in patients with colorectal cancer.

## Figures and Tables

**Table 1:** Basic characteristics of study population.

Characteristics	Trial group (*n*, %) (*n* = 60)	Control group (*n*, %) (*n* = 60)	*P* value
Age (years)			
≥50	10 (16.7)	7 (11.7)	0.83
<50	50 (83.3)	53 (88.3)	
Gender			
Male	43 (71.7)	40 (66.7)	0.55
Female	17 (28.3)	20 (33.3)	
Performance status			
0	34 (56.7)	29 (48.3)	0.36
1,2	26 (43.3)	31 (51.7)	
Location of primary tumor			
Colon	32 (53.3)	34 (56.7)	0.74
Rectum	28 (46.7)	26 (43.3)	
Histological differentiation			
Well/moderately	45 (75.0)	41 (68.3)	0.41
Poorly/unknown	15 (25.0)	19 (31.7)	
Sites of distant metastasis			
Liver	12 (20.0)	10 (16.7)	0.74
Lung	8 (13.3)	8 (13.3)	
Liver and lung	4 (6.7)	3 (5.0)	
Others	0	1 (1.7)	

**Table 2:** Incidence of oxaliplatin-induced neurotoxicity in the trial group and the control group.

Neurotoxicity	Trial group (*n*, %) (*n* = 60)	Control group (*n*, %) (*n* = 60)	*P* value
After two cycles			
Grade 0	52 (86.7)	48 (80.0)	0.04
Grades 1-2	8 (13.3)	11 (18.3)	
Grades 3-4	0 (0.0)	1 (1.7)	
After four cycles			
Grade 0	43 (71.7)	34 (56.7)	0.05
Grades 1-2	14 (23.3)	18 (30.0)	
Grades 3-4	3 (5.0)	8 (13.3)	
After six cycles			
Grade 0	29 (48.3)	18 (30.0)	0.04
Grades 1-2	24 (40.0)	23 (38.3)	
Grades 3-4	7 (11.7)	19 (31.7)	

**Table 3:** Objective tumor response in the trial group and the control group.

Group	*n*	CR (%)	PR (%)	SD (%)	PG (%)	*P* value
Trial group	60	2 (3.3)	24 (40.0)	28 (46.7)	6 (10)	0.72
Control group	60	2 (3.3)	19 (31.7)	32 (53.3)	7 (11.7)	

CR: complete response; PR: partial response; SD: stable disease; PG: progression.

**Table 4:** Adverse events in the trial group and the control group.

Adverse events	Trial group (*n*, %) (*n* = 60)	Control group (*n*, %) (*n* = 60)	*P* value
Anemia			
Grades 1-2	7 (11.7)	8 (13.3)	0.78
Grades 3-4	0	0	
Neutropenia			
Grades 1-2	14 (23.3)	13 (21.7)	0.92
Grades 3-4	7 (11.7)	6 (10.0)	
Thrombocytopenia			
Grades 1-2	10 (16.7)	9 (15.0)	0.80
Grades 3-4	0	0	
Nausea			
Grades 1-2	18 (30.0)	20 (33.3)	0.69
Grades 3-4	0	0	
Vomiting			
Grades 1-2	14 (23.3)	16 (26.7)	0.67
Grades 3-4	0	0	
Diarrhea			
Grades 1-2	12 (20.0)	13 (21.7)	0.56
Grades 3-4	1 (1.7)	3 (5.0)	
Stomatitis			
Grades 1-2	12 (20.0)	11 (18.3)	0.81
Grades 3-4	0	0	
